# Beyond Life Style Interventions in Type 2 Diabetes

**DOI:** 10.1590/1518-8345.0000.2765

**Published:** 2016-11-28

**Authors:** Maria da Graça Pereira

**Affiliations:** Associate External Editor of Revista Latino-Americana de Enfermagem, Associate Professor with Aggregation of Department of Applied Psychology, Escola de Psicologia, Universidade do Minho, Braga, Portugal. E-mail: gracep@psi.uminho.pt

**Figure:**
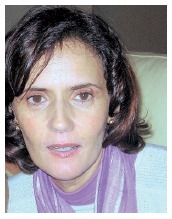

Diabetes is a serious public health problem. Every six seconds, two people are diagnosed with diabetes, and one person dies from diabetes-related causes, in the world. Diabetes is also responsible for more than a million amputations each year across the globe and it is expected that rates, mortality increases 25% in the next decade and 3/4 of people with diabetes live in low and middle income countries^1^.

Initially, type 2 diabetes impacted more the rich countries, but with globalization it reached all continents. Adherence to self-care behavior, in type 2 diabetes is associated with social economic factors. In fact, individuals with lower income and less education are two to four times more likely to develop diabetes and tend to have poorer glycemic control, more diabetes complications, and higher mortality. Lack of access to health care is an important risk factor for the consequences of diabetes among the socioeconomically deprived. The poor are more likely to experience inequality of care once diabetes has developed despite health insurance coverage.

A recent systemic review studying at risk individuals with "pre-diabetes" showed that lifestyle interventions are the primary target to control type 2 diabetes recommended for both the prevention and treatment. But there are major impediments to strategies that can improve healthy lifestyles, at the societal level. For instance, healthy eating is an important part of managing diabetes as well as accessing programs that promote physical exercise. However, globally, there is a shortage in the supply of fruits and vegetables based on a recommended intake of at least ﬁve daily portions, as high as 58% in low-income countries^2^. 

Another recommendation for patients with hyperglycemia is to manage their stress. Chronic stress and ad social isolation, particularly in the elderly, are associated with an increase of glucose levels and diabetes complications. The physical manifestation of chronic stress leads to increased blood pressure, cortisol, and blood glucose levels. Poor income families are exposed to chronic stress, over time, trying to make ends meet that eventually may lead to high levels of cortisol that directly affects the ability of the body to use insulin. 

Diabetes-self management education studies report only short-term improvements in clinical outcomes, so one must wonder why the health behavior paradigm has not taken into consideration the social and economic factors such as access to healthy foods, exercise programs, as well as psychological variables such as family involvement, social and partner support that may predict adverse medical outcomes just as much as the traditional medical risk factors.

Besides policies to improve the interaction between providers and patients, public and economic policies are needed, particularly environmental policies that provide safe places to practice exercise, affordable healthy foods, as well as psychological health for individuals and families, that include assistance regarding the stress towards diabetes, depression, and coping strategies helping the patient to control the illness and promoting adherence to self-care behaviors and quality of life. In a recent meta-analysis of randomized controlled trials Ismail et al^3^ found that type 2 diabetes patients who received behavioral-based diabetes education or psychological interventions showed improvements in both glycemic control and psychological distress.

Interdisciplinary interventions that bring together agencies and organizations responsible for schools, housing, and safety are needed besides lifestyle interventions. Although biomedical and behavioral approaches to health are important and may even be profitable for certain groups in our society, a system approach is needed to deal with the multidimensional aspects related to type 2 diabetes if one wants to curb the actual diabetic epidemic.
